# A Comparison of Acute Pharmacological Effects of Methylone and MDMA Administration in Humans and Oral Fluid Concentrations as Biomarkers of Exposure

**DOI:** 10.3390/biology10080788

**Published:** 2021-08-17

**Authors:** Lourdes Poyatos, Esther Papaseit, Eulalia Olesti, Clara Pérez-Mañá, Mireia Ventura, Xoán Carbón, Marc Grifell, Francina Fonseca, Marta Torrens, Rafael de la Torre, Magí Farré

**Affiliations:** 1 Department of Clinical Pharmacology, Hospital Universitari Germans Trias i Pujol and Institut de Recerca Germans Trias i Pujol (HUGTiP-IGTP), 08916 Badalona, Spain; lpoyatos@igtp.cat (L.P.); cperezm.mn.ics@gencat.cat (C.P.-M.); mfarre.germanstrias@gencat.cat (M.F.); 2 Department of Pharmacology, Therapeutics and Toxicology, Universitat Autònoma de Barcelona (UAB), 08193 Cerdanyola del Vallés, Spain; 3 Integrative Pharmacology and Systems Neuroscience Research Group, Neurosciences Research Program, Hospital del Mar Medical Research Institute (IMIM), 08003 Barcelona, Spain; eulaliaom@gmail.com (E.O.); rtorre@imim.es (R.d.l.T.); 4 Department of Experimental and Health Sciences, Universitat Pompeu Fabra (CEXS-UPF), 08003 Barcelona, Spain; 5 Energy Control, Associació Benestar i Desenvolupament, 08041 Barcelona, Spain; mireia@energycontrol.org (M.V.); nps@energycontrol.org (X.C.); marcgrifellguardia@gmail.com (M.G.); 6 Department of Psychiatry and Forensic Medicine, Universitat Autònoma de Barcelona (UAB), 08193 Cerdanyola del Vallés, Spain; mffonseca@parcdesalutmar.cat (F.F.); mtorrens@parcdesalutmar.cat (M.T.); 7 Institut de Neuropsiquiatria i Adiccions (INAD), Parc de Salut Mar, 08003 Barcelona, Spain

**Keywords:** methylone (3,4-methylenedioxymethcathinone), MDMA (3,4-methylenedioxymethamphetamine), new psychoactive substances (NPS), synthetic cathinones, bath salts, psychostimulants

## Abstract

**Simple Summary:**

Methylone is a synthetic cathinone that is usually used as a substitute for conventional psychostimulants, such as MDMA. Chemically, methylone is considered the β-keto analogue of MDMA, with which it presumably shares similar pharmacological effects. To date, the available data about the human pharmacology of methylone in humans are very scarce and are mainly derived from user experiences, published in internet forums or intoxication reports. Thus, an observational–naturalistic study was conducted to evaluate the acute pharmacological effects and determine biomarkers of exposure in oral fluid of methylone after oral self-administration in comparison to MDMA. Methylone induced the prototypical psychostimulant and empathogenic effects commonly associated with MDMA, although they were of lower intensity. Oral fluid concentrations of methylone can be considered a suitable biomarker of acute exposure, and oral fluid has been proven to be a useful biological matrix of detection.

**Abstract:**

Considered the β-keto analogue of 3,4-methylenedioxymethamphetamine (MDMA, ecstasy), 3,4-Methylenedioxymethcathinone (methylone) is a synthetic cathinone. Over the years, methylone has been used as a substitute for conventional psychostimulants, such as MDMA. To date, little is known about the human pharmacology of methylone; the only available information has been provided by surveys or published intoxication reports. In the present observational–naturalistic study, we evaluate the acute subjective and physiological effects of methylone after oral self-administration in comparison to MDMA in healthy poly-drug users. Fourteen participants (10 males, 4 females) selected their single oral doses of methylone from 100 to 300 mg (*n* = 8, mean dose 187.5 mg) or MDMA from 75 to 100 mg (*n* = 6, mean dose 87.5 mg) based on their experience. Study variables were assessed at 0, 1, 2, and 4 h (h) and included vital signs (non-invasive blood pressure, heart rate, cutaneous temperature) and subjective effects using visual analogue scales (VAS), the 49-item Addiction Research Centre Inventory (ARCI) short form, and the Evaluation of the Subjective Effects of Substances with Abuse Potential (VESSPA-SSE) questionnaire. Additionally, oral fluid concentrations of methylone and MDMA were determined. Acute pharmacological effects produced by methylone followed the prototypical psychostimulant and empathogenic profile associated with MDMA, although they were less intense. Methylone concentrations in oral fluid can be considered a useful biomarker to detect acute exposure in oral fluid. Oral fluid concentrations of MDMA and methylone peaked at 2 h and concentrations of MDMA were in the range of those previously described in controlled studies. Our results demonstrate that the potential abuse liability of methylone is similar to that of MDMA in recreational subjects.

## 1. Introduction

Over the last few years, new psychoactive substances (NPS) have become a trend among substance users seeking non-illegal alternatives to classical illicit drugs. For this reason, these substances are also known as “legal highs”, although, in the market, they are also advertised as bath salts, plant foods, or fertilizer, and are labelled as “not for human consumption” to bypass regulations [1,2]. For the first time, in 2019, synthetic cathinones were one of the most frequently reported groups of NPS to the European Union Early Warning System according to the European Monitoring Centre for Drugs and Drug Addiction (EMCDDA) [3]. These synthetic substances are chemically related to cathinone, a compound with psychostimulant effects, found in the khat plant (*Catha edulis*) [4,5]. In the last decade, numerous new synthetic cathinone derivatives have emerged given the high dynamism of the NPS market. Some of the most well-known derivatives are methylone (3,4-methylenedioxymethcathinone), mephedrone (4-methylmethcathinone), and MDPV (3,4-methylenedioxypyrovalerone).

Methylone, also known as MDMC, M1, and bk-MDMA, is a ring-substitute β-keto analogue of the well-known 3,4-methylenedioxymethamphetamine (MDMA, ecstasy) [6]. Since its first appearance as a “room odorizer” in smartshops, methylone gained popularity as a substitute for the traditional MDMA [7]. Methylone resembles MDMA in its mechanism of action, as methylone acts on the monoaminergic system, inducing the reversal or inhibiting the activity of monoamine reuptake transporters. These actions on the monoaminergic system result in increased extracellular brain levels of monoamines, such as dopamine, norepinephrine, and, mainly, serotonin [8,9,10,11,12]. In this area, there are some discrepancies regarding the potency of its effects on the monoaminergic transporters. Results from an in vitro study suggested that methylone had a selectivity comparable to that of mephedrone and MDMA but displayed a lower potency on transporter-mediated release [8]. Another study concluded that methylone was the most potent serotonin and dopamine uptake inhibitor compared to mephedrone and butylone [13]. Methylone and other cathinones, such as mephedrone, butylone, and ethylone, act as nonselective monoamine uptake inhibitors, similar to cocaine, and have effects on serotonin release similar to MDMA [14]. In general, methylone acts on monoaminergic transporters with potency and selectivity comparable to that of MDMA [8]. Methylone and other cathinones also can activate 5-HT2A receptors and increase extracellular dopamine [13].

Methylone is metabolized in the liver, principally by the enzymatic activity of CYP2D6, located in cytochrome P450, with a limited contribution of CYP1A2, CYP2B6, and CYP2C19 [15]. Similarly to MDMA, its metabolism results in the formation of O-demethylenated metabolites (HHMC, 3,4-methylenedioxy-N-methylcathinone; HMMC, 4-hydroxy-3-methoxy-Nmethylcathinone) and an N-demethylated metabolite (MDC, 3,4-methylenedioxycathinone) [16,17]. The activity of methylone seems to be related to brain concentrations of methylone and MDC but not its hydroxylated metabolites [18].

Whereas MDMA is the fourth most commonly used recreative substance worldwide according to the UNODC, information about methylone prevalence is scarce. Globally, an estimate of 20.5 million people reported use of MDMA in the last year in 2018 [19]. In the European Union, approximately 2.7 million people aged 15–64 were estimated to have used MDMA in the previous year [3]. On the other hand, the use of methylone as an adulterant and its rebranding as other psychostimulant substances hinder the determination of its prevalence of use, since this unintentional use is not reflected in surveys [20,21]. Most of the information regarding the prevalence of the use of methylone comes from seizure data or reported intoxications. According to the National Forensic Laboratory Information System (NFLIS) data, methylone was the most reported synthetic cathinone (33.35%) in the USA between 2013 and 2015 [22]. 

Similarly to other synthetic cathinones, methylone can be administered via different routes, including oral, intranasal (insufflation), intravenous, sublingual, and rectal administration. The most common route is oral consumption of tablets or pills containing methylone. In accordance with recreative drug user reports, doses up to 100 mg are considered to be low, doses from 100 mg to 200 mg are moderate, and doses above 200 mg are considered to be high. After oral administration of methylone, users described the onset of effects at 15–60 min after administration, with peak effects occurring at 60–90 min and a total duration of effects of 3–5 h [23]. A frequent pattern of use, also similar to other cathinones, is to firstly administer a large dose followed by smaller re-doses in order to extend the effects [23,24]. 

According to user reports, methylone also displays a similar but milder range of effects compared to MDMA that encompasses stimulation, calm euphoria, a sense of well-being and happiness, alertness, reduced fatigue, heightened empathy, and entactogenic effects (sense of oneness) [25,26]. Among the published cases of intoxication involving methylone [27,28,29,30,31,32], a patient that visited the emergency department after using 1.0–1.5 g of methylone presented vomiting, palpitations, agitation, sweating, paresthesia, muscle twitching, tremors, and vertigo [33]. Other adverse effects associated with methylone intoxication are hyperthermia, anxiety, seizures, psychosis, hallucinations, and suicidal ideation [6].

Oral fluid concentrations of amphetamine derivatives are considered a suitable biomarker for the detection of their acute use, and they have been useful in cases of intoxication and driving under the influence of substances [34,35]. In the case of MDMA, there is evidence of a correlation between the oral fluid and blood concentrations [36,37]. However, there are no previous reports evaluating the possible usefulness of methylone oral fluid concentrations as a biomarker of acute exposure.

To date, little is known about the pharmacokinetic and pharmacodynamic profile of methylone in humans. The purpose of our observational study was to assess the acute pharmacological effects and oral fluid concentrations of methylone as a biomarker of exposure in recreational users after oral administration in a naturalistic environment. The subjective and physiological effects and oral fluid concentrations of methylone are compared to those of its non-β-analogue MDMA, which was also administered in similar conditions.

## 2. Materials and Methods

### 2.1. Participants

Fourteen healthy volunteers were included (10 males and 4 females). The subjects were recreative drug users that had previous experience with MDMA and/or synthetic cathinones at least once in their lifetime. Exclusion criteria included a history of any serious medical or mental disorder, including substance use disorder (except nicotine), serious adverse reactions to MDMA and/or synthetic cathinones, and use of chronic medication. 

Participants were recruited by word-of-mouth through Energy Control. The protocol of this study was approved by the Parc de Salut Mar Clinical Research Ethics Committee (ref. 2016/6700/I). The study was conducted in accordance with the Declaration of Helsinki and Spanish legislation. All participants were fully informed of the purpose and procedures of the study and they were provided with a written informed consent form before enrolling in the study.

### 2.2. Design and Treatments

The study was designed as a non-controlled prospective observational study in a naturalistic setting with methylone and MDMA self-administration. The methodology, including procedures and evaluations, coincides with previous observational–naturalistic studies aimed at evaluating the acute effects of other NPS [38,39,40]. Six subjects (5 M, 1 F) self-administered MDMA and 8 subjects (5 M, 3 F) self-administered methylone. Each subject participated only in one session. Participants brought their own substance obtained from an unknown source, which was tested by Energy Control, a harm reduction organization that provides a drug checking service to drug users. Pills containing methylone and MDMA were analyzed by gas chromatography associated with mass spectrometry (GC/MS), a technique that traces the presence of the most frequent drugs of abuse, such as MDMA, cocaine, heroin, amphetamine and methamphetamine, LSD, and multiple NPS (methylone, mephedrone, and other synthetic cathinones, synthetic cannabinoids, tryptamines, among others). The testing of the pills showed a more than 95% purity of methylone and MDMA, as well as the absence of toxic components or adulterants [38,39,40]. 

In both sessions, participants selected the dose of methylone or MDMA according to their previous drug use experience. Doses of methylone could be selected from a range (75–300 mg) that was previously defined according to the consulted literature [23]. The WHO Expert Committee on Drug Dependence (Thirty-Sixth Meeting, Geneva, Switzerland, 16–20 June 2014) established that 60 mg of methylone was the threshold dose and doses over 250 mg were often related to very strong activity [23]. Some users have reported uses of 300 mg of methylone as a common dose [www.erowid.org] (accessed on 15 June 2021). The mean of the selected doses of MDMA was 87.50 mg (3 subjects ingested 75 mg (2 males and 1 female), 3 males 100 mg). In the other study session, methylone doses ranged from 100 to 300 mg, with a mean of 187.50 mg. Based on their dose selection, 1 male ingested 100 mg, 2 subjects 150 mg (1 male and 1 female), 4 subjects 200 mg (2 males and 2 females), and 1 male 300 mg. 

### 2.3. Procedures

Sessions were conducted in a private club closed to the public for the study, where participants were summoned at 15:00 h and stayed until the end of the session at 20:00 h. Upon arrival, urine samples were collected to detect the presence of any conventional drug (benzodiazepines, barbiturates, morphine, cocaine, amphetamines, methamphetamine, MDMA, marijuana, phencyclidine) with Instant-View, Multipanel 10 Test Drug Screen (Alfa Scientific Designs Inc., Poway, CA, USA). Subjects were not allowed to use any recreational drug 2 days prior to the session or consume alcohol and caffeinated beverages in the previous 24 h. Participants received instructions and training on the procedures and questionnaires used throughout the sessions. 

The sessions were conducted in a naturalistic setting. Participants were allowed to talk, read, listen to music, or play games, except during the evaluation times. However, they were asked to refrain from talking about the effects of the substance. All the subjects were evaluated simultaneously at baseline, 1, 2, and 4 h after administration of methylone or MDMA, which occurred approximately at 16:00 h. At each time point, evaluations were followed in a specific order: physiological effects, oral fluid collection, and subjective effects scales and questionnaires.

### 2.4. Physiological Effects

Non-invasive systolic blood pressure (SBP), diastolic blood pressure (DBP), and heart rate (HR) were measured with subjects in the sitting position, using an automatic Omron monitor at baseline, 1, 2, and 4 h (h) after self-administration. Cutaneous temperature was determined at the same time points.

### 2.5. Subjective Effects

Subjective effects were evaluated at baseline, 1, 2, and 4 h after self-administration using visual analogue scales (VAS), the Addiction Research Center Inventory (ARCI), and the Evaluation of Subjective Effects of Substances with Abuse Potential questionnaire (VESSPA-SSE). 

Visual analogue scales (100 mm, from “not at all” to “extremely”) contained 30 items that subjects were asked to rate, such as: intensity, stimulated, high, good effects, liking, happiness, drunkenness, changes in colors, changes in shapes, changes in lights, hallucinations (seeing lights or spots), hallucinations (seeing of animals), changes in hearing, hallucinations (hearing sounds or voices), different or changed body feeling, unreal body feeling, changes in distances, different surroundings, unreal surroundings, confusion, fear, depression or sadness, drowsiness, dizziness, bad effects, headache, sickness, vertigo, shortness of breath, and face flushing [38,39,40]. 

The standardized ARCI 49-item short form is a true/false questionnaire used to evaluate the subjective effects of drugs of abuse. This inventory includes five subscales that assess pentobarbital–chlorpromazine–alcohol-like effects (PCAG, sedation), morphine–benzedrine-like effects (MBG, euphoria), lysergic acid diethylamide-like effects (LSD, dysphoria), benzedrine-like effects (BG, intellectual efficiency), and amphetamine-like effects (A, increased energy) [38,39,40]. 

The VESSPA-SSE is a questionnaire sensitive to subjective effects related to stimulants such as MDMA that includes six subscales: sedation (S), psychosomatic anxiety (ANX), changes in perception (CP), pleasure and sociability (SOC), activity and energy (ACT), and psychotic symptoms (PS) [38,39,40].

### 2.6. Oral Concentrations

In both sessions, oral fluid samples were collected with Salivette to determine methylone and MDMA concentrations in oral fluid at baseline, 1 h, 2 h, and 4 h. All samples were centrifuged after collection and stored frozen at −20 °C until analysis. Methylone and MDMA oral fluid concentrations were quantified via liquid chromatography tandem–mass spectrometry (LC–MS/MS) [38,39,40].

### 2.7. Statistical Analysis 

The determination of the sample size was based on the methodology of bioequivalence studies, which resulted in 5–6 subjects needed, considering an alpha risk of 0.05, a power of 80%, with a difference of at least 35% between MDMA to methylone in the intensity/high effect and with 20% of variability.

Vital signs (SBP, DBP, and HR) and subjective effects (VAS, ARCI, and VESSPA-SSE) were baseline-adjusted. Maximum effects and the time in which maximum effects appeared were determined, and the area under the curve (AUC_0–4h_) was calculated with the trapezoidal rule. 

For oral fluid concentrations of MDMA and methylone, only a descriptive analysis was presented showing main pharmacokinetics data such as the maximum concentration (Cmax), the time required to reach maximum concentrations (Tmax), and AUC_0–4h_. These parameters were calculated using the Pharmacokinetic Functions for Microsoft Excel (Joel Usansky, Atul Desai, and Diane Tang-Liu, Department of Pharmacokinetics and Drug Metabolism, Allergan, Irvine, CA, USA). 

The following stages in the statistical analysis comprised 4 tests. Firstly, a two-way ANOVA with dose and gender as factors was conducted to determine whether the difference in doses or gender had an impact on the acute effects of methylone or MDMA. In the case of methylone, out of all variables analyzed, only 17 out of 131 variables that corresponded to effects with low scores were found to be significant. For MDMA, none of the variables were significant. Thus, given that any of the main effects associated with methylone or MDMA showed significant differences related to dose and gender, all participants were grouped independently of these factors considering just one group of methylone and MDMA.

Secondly, the comparison of Emax and AUC_0–4h_ values of physiological and subjective effects between MDMA and methylone was performed with an independent samples *t*-test. Tmax values were compared with the non-parametric Mann–Whitney *U*-test. 

Thirdly, to find possible significant changes from baseline, a Dunnett multiple comparison test was conducted to compare each time point with baseline in both drug conditions (0–1 h, 0–2 h, 0–4 h). 

Finally, to compare the time-course of all the pharmacological effects between methylone and MDMA, they were evaluated with a two-way ANOVA test with time and drug condition as factors. When these results were significant, a Tukey post-hoc test compared the differences in each time point between conditions. 

Statistical analysis was carried out using PASW Statistics version 18 (SPSS Inc., Chicago, IL, USA). Differences were considered statistically significant when the resulting *p* value was <0.05.

## 3. Results

Table 1 provides a summary with the statistically significant results (Emax, Tmax, AUC_0–4h_) of the physiological and subjective effects after methylone and MDMA self-administration. Oral fluid concentrations of both substances are presented in Table 2.

### 3.1. Participants

In total, 14 subjects (10 males, 4 females) were selected to participate in the study. Eight subjects (5 males, 3 females) were included for the self-administration of methylone. Participants had a mean age of 30 ± 5 years (range 23–37), weighed 64.88 ± 9.20 kg (range 54.0–78.0), and had a mean body mass index (BMI) of 22.24 ± 3.50 kg/m^2^ (range 16.48–26.03). The mean dose of methylone was 187.50 ± 58.25 mg (range 100–300), which, adjusted to weight, resulted in 2.97 ± 0.99 mg/kg (range 1.28–4.35). At the beginning of the session, urine samples were collected to determine previous drug use. All subjects tested negative in the urine drug tests. 

Six participants (5 males, 1 female) were included for the self-administration of MDMA. Participants had a mean age of 29 ± 6 years (range 22–38), weighed a mean of 65.33 ± 8.45 kg (range 54.0–75.0), and had a mean BMI of 22.41 ± 3.17 kg/m^2^ (range 16.48–25.65). The mean dose of MDMA was 87.50 ± 13.69 mg (range 75–100), which, adjusted to weight, resulted in 1.35 ± 0.19 mg/kg (range 1.00–1.54). Five subjects obtained negative results in the urine test and one subject tested positive for cannabis. This participant reported that their last cannabis use was 48 h prior to the session, as specified in our selection criteria. 

In both cases, all selected participants reported previous experience with psychostimulants (including MDMA, amphetamines, NPS/synthetic cathinones, cocaine), cannabis, and hallucinogens. See Table S1 for history of drug use.

### 3.2. Physiological Effects

**Figure 1:**
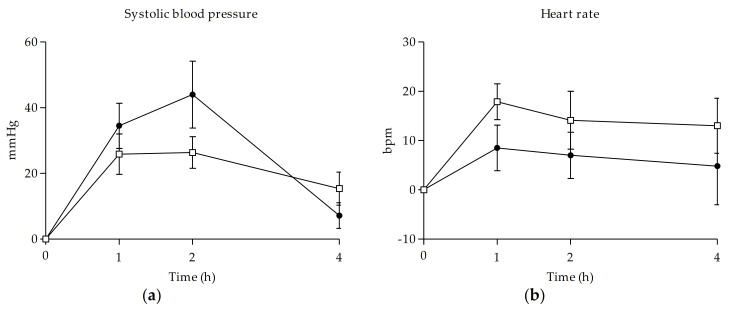
Time course of systolic blood pressure (**a**) and heart rate (**b**) after methylone and MDMA oral administration. (□, 100–300 mg methylone (*n* = 8); ●, 75–100 mg MDMA (*n* = 6); mean, standard error).

With respect to physiological effects, both methylone and MDMA produced a statistically significant increase in SBP and DBP compared to baseline over the first 2 h, although this significant effect was prolonged to 4 h in the case of methylone (see Figure 1). Regarding HR, only methylone showed a significant increase in the first hour. Neither of the substances caused significant variations in temperature. 

In addition, the maximum effects on SBP and BDP were higher after the administration of MDMA, whereas methylone showed higher maximal effects on HR (see Table 1). However, no significant differences between methylone and MDMA were detected in Emax, AUC_0–4h_, and Tmax of the cardiovascular effects.

### 3.3. Subjective Effects 

Methylone and MDMA produced significant subjective effects, which were collected in VAS, ARCI, and VESSPA-SSE. Overall, subjects reported subjective effects starting at 1 h, with maximum values ranging from 1 h to 2 h; hence, most of these effects had almost disappeared at 4 h. 

**Figure 2:**
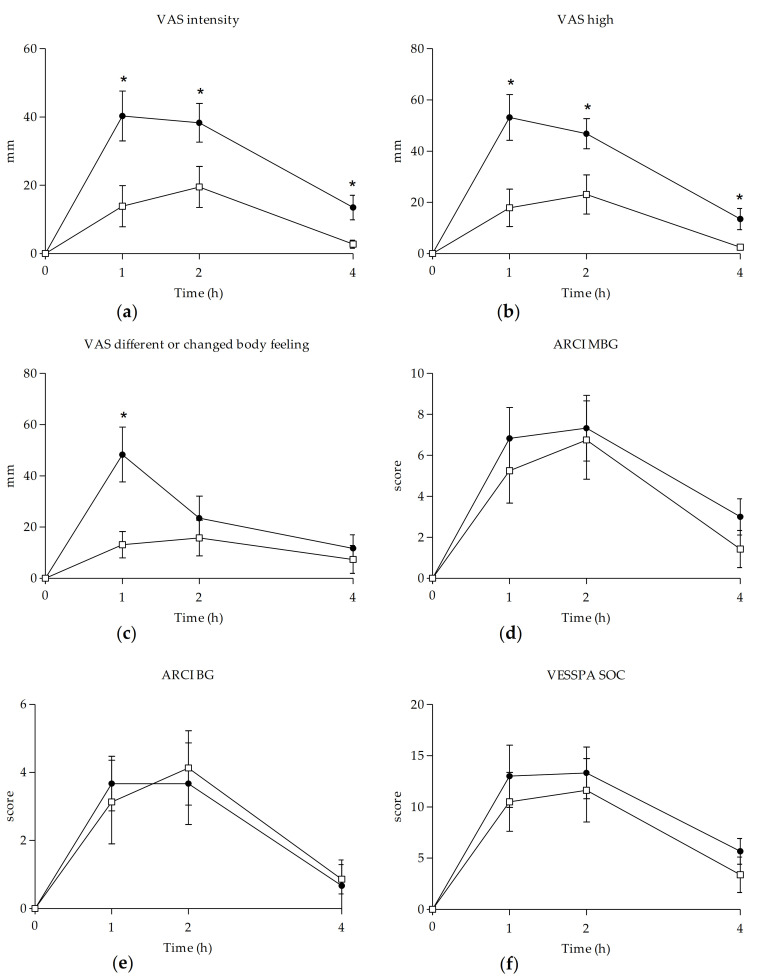
Summary of the time course of subjective effects collected through VAS (intensity (**a**), high (**b**), different or changed body feeling (**c**)), ARCI (MBG (euphoria) (**d**), BG (intellectual efficiency) (**e**)), and VESSPA-SSE (SOC (pleasure and sociability) (**f**)) questionnaires after methylone and MDMA oral administration. (□, 100–300 mg methylone (*n* = 8); ●, 75–100 mg MDMA (*n* = 6); mean, standard error). Statistical differences of *p* < 0.05 between conditions are indicated with “*”. See text for abbreviations.

When compared to baseline, both substances caused significant changes in VAS measures, reflecting stimulant-like effects (“intensity”, “stimulated”, “high”, “good effects”, “liking”, “content”, “drunkenness”), changes in perception (“changes in lights” (methylone), “different or changed body feeling” (MDMA), “different surroundings” (MDMA)), and face flushing. Subjects also mentioned slight feelings of dizziness and headache after methylone and MDMA administration (see Table 1 and Figure 2) [38,39,40]. When comparing both conditions, marked differences were detected in maximum effects, AUC_0–4h_, and at several T-C points in scales related to stimulant-like effects and body perception, with higher values after MDMA administration. 

Regarding the ARCI questionnaire, significant differences from baseline were detected between substances for subscales MBG (euphoria), BG (intellectual efficiency and energy), and A (amphetamine). Subjects who self-administered methylone also reported significant changes in the PCAG subscale (sedation). When comparing methylone and MDMA, peak scores in MBG and BG subscales were very similar, although with significantly earlier onset (Tmax) after MDMA administration (see Table 1 and Figure 2). However, no statistical differences in maximal effects, AUC_0–4h_, and T-C points in any of the subscales were observed. 

In relation to VESSPA-SSE, methylone and MDMA produced significant changes compared to baseline in some subscales, such as S (sedation), ANX (anxiety), SOC (pleasure and sociability), and ACT (activity and energy). The most relevant effects caused by both conditions with the highest scores of maximum effects were SOC and ACT. However, no statistically significant differences were found in peak effects, AUC_0–4h_, or T-C points between the two substances in any of the subscales (see Table 1 and Figure 2). The only significant difference was the time of maximum values for the ANX subscale, which showed an earlier onset for MDMA.

The selected doses of both substances were well-tolerated, and no serious adverse effects appeared.

### 3.4. Oral Fluid Concentrations

**Figure 3:**
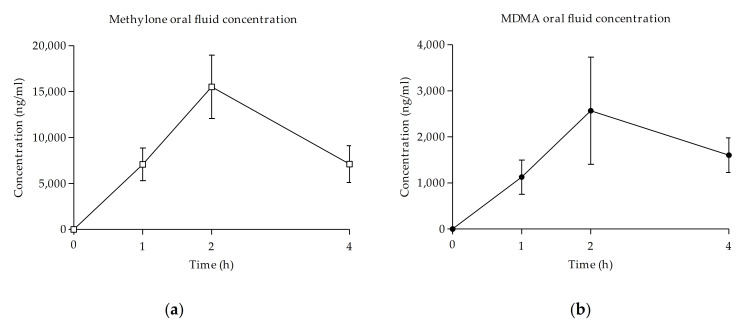
Evolution of concentrations of methylone (**a**) and MDMA (**b**) in oral fluid over time (□, 100–300 mg methylone (*n* = 8); ●, 75–100 mg MDMA (*n* = 6); mean, standard error).

Oral fluid concentrations of methylone increased rapidly until maximum concentrations were reached at 2 h, with a mean Cmax of 15,514.00 ± 9748.86 ng/mL. The AUC_0–4h_ obtained from the concentrations was 40,623.79 ± 20,001.70 ng/mL·h. Concentrations of methylone started to rapidly decrease at 4 h (see Table 2 and Figure 3).

In the case of MDMA, oral fluid concentrations increased until they reached their peak at 2 h after administration in all the subjects, with a mean Cmax of 2936.37 ± 2761.57 ng/mL. MDMA obtained an AUC_0–4h_ of 6586.44 ± 5229.92 ng/mL·h. Concentrations of MDMA in oral fluid started to decrease at 4 h (see Table 2 and Figure 3). 

In both cases, subjects ended the sessions with remaining concentrations of methylone or MDMA in oral fluid.

## 4. Discussion

To the best of our knowledge, this is the first observational study that evaluates the acute physiological and subjective effects of methylone in humans and compares its pharmacological profile with MDMA. Moreover, the other purpose of this study was to determine oral fluid concentrations of methylone and see how they relate to the time course of the pharmacological effects. 

Our main finding is that the oral administration of methylone in a naturalistic setting exhibits prototypical psychostimulant and empathogenic effects in healthy and experienced recreational drug users. Methylone and traditional MDMA showed similar pharmacological effects. 

Methylone and MDMA produced perceptible increases in SBP and DBP, with higher effects after MDMA administration, although differences between them were not statistically significant. Interestingly, maximum effects in SBP occurred earlier in time after methylone intake (1.5 h), and this rise was also further extended in time (4 h) compared to MDMA, which caused higher effects at 2 h that returned to baseline values at the end of the session. Regarding HR, methylone induced a higher increase than MDMA at 1 h after administration. These findings are consistent with previous methylone intoxication reports which described tachycardia and hypertension as some of the clinical manifestations [41]. In the case of MDMA, cardiovascular effects are in line with previous studies under controlled conditions that reported marked increases in SBP, DBP, and HR [42,43,44,45]. 

As expected, methylone and MDMA displayed prototypical psychostimulant and empathogenic effects, extensively described for MDMA [42,43,44,45,46,47,48,49]. In general, subjective effects appeared in the first hour in both conditions and reached maximum values at 2 h after methylone administration, whereas most of these effects peaked earlier, at 1.5 h, in the case of MDMA administration. This finding slightly differs from user reports, which define maximum effects at 1 or 1.5 h after methylone administration [23]. Methylone produced increases in VAS related to stimulation and well-being (stimulation, high, content, good effects), although these effects were half as intense as those produced by MDMA. A possible explanation for these differences could be that the doses selected are not comparable, meaning that methylone was underdosed; however, the tested doses were similar to those most frequently selected by habitual users. For this reason, our data suggest that common doses of methylone produce similar subjective effects to MDMA, although they are milder. In relation to the effects related to perceptual alterations, subjects under methylone influence did not report marked differences, contrary to those participants that self-administered MDMA, who experienced significant changes in body feeling and surroundings. 

The profile of physiological and subjective effects produced by methylone in our naturalistic setting was in line with preliminary data obtained from a dose-finding study administering oral doses of 50–150 mg of methylone in a controlled environment [50]. 

In relation to the effects obtained through ARCI and VESSPA-SSE, methylone showed a similar profile to MDMA and other psychostimulant substances such as amphetamines and mephedrone. Methylone scored predominantly in ARCI subscales related to euphoria (MBG), intellectual efficiency and energy (BG), and in amphetamine-like effects (A). In the same manner, VESSPA-SSE results reflect an increase in activity and energy (ACT) and pleasure and sociability (SOC). However, those stimulant effects usually sought by recreative users also coexisted with sedation and anxiety. Overall, coinciding with previous user reports, most of the subjective effects exhibited by methylone disappeared 4 h after administration [44]. 

As previously mentioned, there is no published information about the pharmacokinetic profile of methylone in humans to use as a comparison with our findings; the only data available come from studies in rodents [51]. According to the results of our analysis, in oral fluid, methylone reached peak concentrations of 15,514.00 ± 9748.86 ng/mL while MDMA obtained maximum levels of 2936.37 ± 2761.57 ng/mL. Overall, concentrations were not comparable given the great difference between conditions. In both sessions, all the subjects reached maximum levels of methylone and MDMA at 2 h after self-administration and decreased at 4 h. 

Our results of the oral fluid concentrations of MDMA are similar to those previously published after the administration of 100 mg of MDMA [36,37] and 1–1.6 mg/kg [52].

Previous studies evaluating concentrations of MDMA in oral fluid and blood reported highly variable oral fluid to blood (OF/B) ratios, with concentrations notably higher in oral fluid. This ratio showed a maximum value of 18.1 ± 7.9 (range 10.3–32.3) at 1.5 h after a 100 mg dose of MDMA [36,37]. Other study evaluating oral fluid and plasma correlation reported a median overall OF/B of 5.2 (range 0.1–40.4) after administering the low dose (1.0 mg/kg) and a OF/B of 6.0 (range 0.4–52.3) following the high dose of MDMA (1.6 mg/kg) [52]. Although oral fluid and blood concentrations exhibited a statistically significant correlation, we cannot confirm that blood concentrations can be predicted from oral fluid concentrations due to the high variability of OF/B ratios [52]. Our MDMA oral fluid concentrations would theoretically correspond to the range of concentrations described in previous studies, obtaining a Cmax in plasma of approximately 205 ng/mL.

Currently, there is no study with synthetic cathinones that examines OF/B ratios. However, previous studies on the oral administration of mephedrone provide independent pharmacokinetic data of oral fluid concentrations and blood concentrations that allow us to estimate the OF/B ratios of mephedrone. After comparing the Cmax values of mephedrone collected from a controlled study and an observational study, OF/B ratios resulted in values of 49.43, 4.28, and 11.73 following oral mephedrone doses of 100, 150, and 200 mg, respectively [40,53]. Using data from a study that compared blood concentrations of mephedrone and MDMA, estimated OF/B ratios were found to be 22.28 after a 200 mg mephedrone dose and 22.40 after a 100 mg MDMA dose [40,44]. There are no previously published results about blood concentrations of methylone in experimental or observational studies. The OF/B ratio of methylone cannot be calculated from our results because of a lack of blood concentrations. When comparing the time course of oral fluid concentrations and acute effects, peak concentrations of methylone coincide with the maximum subjective effects, which also appeared at 2 h. Although it is still unclear whether oral fluid concentrations fully correlate with those in blood, this non-invasive sample collection was considered the most suitable for the design of the experiment as an observational study in a naturalistic environment. These results also demonstrate that oral fluid concentrations are a suitable, non-invasive, alternative biomarker that can be used to identify acute methylone use. 

Additionally, this observational study also provided unique preliminary data about the acute effects and oral fluid concentrations of mephedrone, another synthetic cathinone closely related to methylone, administered by oral and intranasal routes [40]. Mephedrone effects were also in line with the typical profile of psychostimulants, although the maximum values of subjective effects after oral administration were higher compared to methylone and showed close similarity to those of MDMA. These results suggest that even though both synthetic cathinones and their non-β-analogue share a clinical profile, the intensity of their effects differs, given that those induced by methylone are milder compared to those of MDMA and mephedrone. The difference observed in methylone and MDMA’s effects can be explained in part by their molecular activity. Methylone has exhibited some affinity for binding 5-HT2A receptors but at significantly lower potencies than MDMA. Methylone has been described as a partial agonist at the 5-HT1A receptor, with weak antagonist effects on 5-HT2C receptors, contrary to MDMA, which is considered a partial agonist rather than an antagonist [54,55]. 

Moreover, mephedrone also produced similar stimulant-like effects to MDMA in controlled conditions, but with a more rapid onset and shorter duration of effects [44]. This would be contrary to our results, which showed that the maximum effects induced by methylone appeared later compared to MDMA. However, further investigations of methylone administration in controlled conditions are required to confirm the findings obtained in this observational study. 

This study has limitations typically associated with observational–naturalistic designs. The study was non-placebo-controlled (negative control) and open-label, since participants selected their doses according to their experience; hence, its design makes it susceptible to an expectancy bias. Moreover, the naturalistic environment could have influenced their subjective reports. Concentrations of methylone and MDMA were only analyzed in oral fluid. Blood samples were not collected in order to maintain the naturalistic setting. Another limitation to consider was the low sample size, which decreased the statistical power of the study. Additionally, sessions were divided into a few time-point evaluations. More evaluations would have allowed us to define a more complete time course of pharmacological effects and oral fluid pharmacokinetics. Finally, subjects were not genotyped for the genetic polymorphism of CYP2D6 involved in methylone and MDMA metabolism, which could have had an impact on the outcomes of the study. 

However, despite its limitations, this design of study is useful and provides valuable information about novel or emergent substances of which there are still no available data in humans. Moreover, the MDMA effects observed in this study are consistent with those described in previous experimental studies administering MDMA [44]. In the same way, this consistency between results obtained from a naturalistic and an experimental study was proven in the administration of mephedrone [40,44] or THC [56,57]. With this in mind, the following strengths of the study should also be considered. Firstly, the sample included participants of both genders. Moreover, subjects were free to select their doses based on their preference and previous experience. All the measurements were taken with validated methodology (questionnaires and rating scales) and determinations were made with validated analytic techniques. The pharmacological effects of methylone were compared with those of a well-known psychostimulant, such as MDMA, which was administered in similar conditions. Finally, the study was conducted in a naturalistic setting, so that the experience mimicked a more recreational scenario compared to controlled studies.

## 5. Conclusions

This observational–naturalistic study constitutes an initial preliminary approach to the determination of the acute effects and oral fluid concentrations of methylone after the oral administration of known doses of methylone. Our findings suggest that the pharmacological effects produced by methylone follow the prototypical psychostimulant and empathogenic profile associated with MDMA, including euphoria, stimulation, alteration of perception, and an increase in energy and sociability. Although the subjective effects were similar, those induced by methylone were less intense and peaked later in time compared to MDMA. Oral fluid concentrations of methylone changed in time following the same pattern as the time course of the acute effects, peaking at 2 h after administration. These results confirm that methylone can be considered a suitable biomarker of exposure and that oral fluid is, in the same way, a useful biological matrix to monitor and detect recent methylone use. Finally, our results suggest that the abuse liability and toxicity of methylone is similar to that of MDMA.

## Figures and Tables

**Table 1:** Summary of the statistically significant results on physiological and subjective effects after methylone (*n* = 8) and MDMA (*n* = 6) self-administration. Only variables with some statistically significant differences in any of the parameters (Emax, Tmax, AUC_0–4h_) and Dunnett’s test are presented.

	Parameters	Mean ± SD		T-Student	Dunnet’s Test	
		Methylone	MDMA	*p* Value	Methylone	MDMA
Physiological effects						
SBP (mmHg)	Emax	31.25 ± 14.77	46.83 ± 20.83	0.126	a, b, c	a, b
	Tmax	1.5 (1.0–4.0)	2.0 (1.0–2.0)	0.659		
	AUC_0–4h_	80.81 ± 42.89	107.67 ± 44.34	0.275		
DBP (mmHg)	Emax	19.63 ± 13.96	32.17 ± 11.29	0.097	a, b, c	a, b
	Tmax	2.0 (1.0–4.0)	2.0 (1.0–2.0)	0.150		
	AUC_0–4h_	55.19 ± 31.02	78.92 ± 30.33	0.178		
HR (bpm)	Emax	20.50 ± 19.78	10.67 ± 18.22	0.360	a	NS
	Tmax	3.0 (1.0–4.0)	3.0 (1.0–4.0)	0.436		
	AUC_0–4h_	52.06 ± 39.16	23.83 ± 38.73	0.205		
Subjective effects						
VAS intensity (mm)	Emax	20.0 ± 16.48	47 ± 11.19	**0.005**	a, b	a, b
	Tmax	2.0 (1.0–2.0)	1.5 (1.0–2.0)	0.411		
	AUC_0–4h_	45.88 ± 43.66	111.33 ± 36.34	**0.012**		
VAS stimulated (mm)	Emax	22.50 ± 18.81	50.17 ± 17.12	**0.015**	b	a, b
	Tmax	2.0 (1.0–4.0)	1.5 (1.0–4.0)	0.160		
	AUC_0–4h_	50.56 ± 47.38	113.17 ± 40.92	**0.024**		
VAS high (mm)	Emax	24.0 ± 21.28	60.17 ± 14.96	**0.004**	a, b	a, b
	Tmax	2.0 (1.0–4.0)	1.5 (1.0–2.0)	**0.032**		
	AUC_0–4h_	55.06 ± 53.54	136.92 ± 41.05	**0.009**		
VAS good effects (mm)	Emax	35.63 ± 30.63	67.83 ± 16.51	**0.039**	a, b	a, b
	Tmax	2.0 (1.0–2.0)	1.5 (1.0–2.0)	0.548		
	AUC_0–4h_	74.06 ± 67.08	167.67 ± 54.01	**0.016**		
VAS content (mm)	Emax	35.25 ± 30.38	74.50 ± 18.96	**0.017**	a, b	a, b, c
	Tmax	1.5 (1.0–2.0)	1.0 (1.0–2.0)	0.133		
	AUC_0–4h_	80.06 ± 82.59	186.25 ± 52.39	**0.018**		
VAS change in lights (mm)	Emax	4.88 ± 4.82	20.00 ± 25.11	0.118	a, b	NS
	Tmax	1.0 (0.0–2.0)	1.5 (0.0–4.0)	0.491		
	AUC_0–4h_	10.88 ± 11.85	40.00 ± 53.35	0.156		
VAS different body feeling (mm)	Emax	22.25 ± 20.60	50.33 ± 22.59	**0.032**	NS	a
	Tmax	2.0 (0.0–4.0)	1.0 (1.0–2.0)	0.258		
	AUC_0–4h_	44.13 ± 43.86	95.25 ± 53.40	0.072		
VAS different surrounding (mm)	Emax	4.38 ± 7.50	17.33 ± 17.68	0.085	NS	a
	Tmax	0.5 (0.0–4.0)	1.0 (0.0–1.0)	0.581		
	AUC_0–4h_	5.69 ± 10.39	27.33 ± 39.71	0.161		
VAS dizziness (mm)	Emax	2.13 ± 2.53	13.00 ± 9.84	**0.010**	a	NS
	Tmax	0.5 (0.0–4.0)	1.0 (0.0–4.0)	**0.034**		
	AUC_0–4h_	2.63 ± 3.02	20.17 ± 20.68	**0.034**		
VAS headache (mm)	Emax	20.25 ± 28.93	5.83 ± 7.68	0.261	c	NS
	Tmax	3.0 (0.0–4.0)	1.0 (0.0–4.0)	0.629		
	AUC_0–4h_	23.63 ± 28.94	10.58 ± 18.86	0.357		
VAS face flushing (mm)	Emax	31.25 ± 21.91	45.00 ± 22.74	0.275	b	a
	Tmax	2.0 (1.0–4.0)	1.5 (1.0–4.0)	0.406		
	AUC_0–4h_	64.50 ± 56.75	89.33 ± 58.31	0.439		
ARCI PCAG (score)	Emax	−1.25 ± 1.58	−0.83 ± 3.54	0.771	a	NS
	Tmax	1.0 (1.0–4.0)	1.0 (1.0–2.0)	0.105		
	AUC_0–4h_	−2.75 ± 3.73	−1.58 ± 8.39	0.730		
ARCI MBG (score)	Emax	7.5 ± 5.13	8.0 ± 3.35	0.839	a, b	a, b
	Tmax	2.0 (1.0–2.0)	1.0 (1.0–2.0)	**0.011**		
	AUC_0–4h_	16.63 ± 13.21	20.83 ±10.05	0.528		
ARCI BG (score)	Emax	4.63 ± 3.34	4.67 ± 2.25	0.979	a, b	a, b
	Tmax	2.0 (1.0–4.0)	1.0 (1.0–2.0)	**0.030**		
	AUC_0–4h_	10.06 ± 7.83	9.83 ± 6.38	0.954		
ARCI A (score)	Emax	5.38 ± 3.29	5.83 ± 1.17	0.752	a, b	a, b, c
	Tmax	1.5 (1.0–4.0)	1.0 (1.0–2.0)	0.106		
	AUC_0–4h_	12.56 ± 7.77	15.75 ± 2.79	0.360		
VESSPA S (score)	Emax	3.38 ± 2.50	2.83 ± 2.23	0.683	b, c	a, c
	Tmax	4.0 (1.0–4.0)	1.0 (1.0–1.0)	0.061		
	AUC_0–4h_	7.06 ± 6.45	5.92 ± 6.09	0.742		
VESSPA ANX (score)	Emax	7.50 ± 4.69	6.67 ± 4.37	0.741	b, c	a, b
	Tmax	3.0 (1.0–4.0)	1.0 (1.0–2.0)	**0.019**		
	AUC_0–4h_	17.94 ± 11.17	16.50 ± 8.82	0.800		
VESSPA SOC (score)	Emax	12.25 ± 8.31	14.50 ± 5.96	0.585	a, b	a, b
	Tmax	2.0 (1.0–2.0)	1.0 (1.0–2.0)	0.298		
	AUC_0–4h_	31.31 ± 25.40	38.67 ± 17.84	0.557		
VESSPA ACT (score)	Emax	9.75 ± 6.25	13.33 ± 4.84	0.268	a, b	a, b
	Tmax	1.0 (1.0–2.0)	1.5 (1.0–2.0)	0.877		
	AUC_0–4h_	24.44 ± 17.87	32.58 ± 15.71	0.393		
VESSPA PS (score)	Emax	2.63 ± 2.20	1.33 ± 1.21	0.221	b	NS
	Tmax	1.0 (0.0–2.0)	2.0 (0.0–2.0)	0.362		
	AUC_0–4h_	5.63 ± 5.58	2.25 ± 2.36	0.192		

Abbreviations: Area under the curve (AUC), visual analogue scales (VAS), Addiction Research Center Inventory (ARCI) (PCAG (sedation), MBG (euphoria), BG (intellectual efficiency), and A (increased energy)), Evaluation of Subjective Effects of Substances with Abuse Potential questionnaire (VESSPA-SSE) (sedation (S), psychosomatic anxiety (ANX), pleasure and sociability (SOC), activity and energy (ACT), and psychotic symptoms (PS)), not significant (NS). Results of Cmax and AUC_0–4h_ are presented as mean ± standard deviation, Tmax is shown as median (min–max). To compare the T-C with baseline values, a post-hoc Dunnett’s test for multiple comparisons was performed. Statistical differences between conditions are indicated as “a” (times 0–1 h), “b” (times 0–2 h), “c” (times 0–4 h). Significant T-Student *p* <0.05 values are marked in bold.

**Table 2:** Concentrations of methylone (*n* = 8) and MDMA (*n* = 6) in oral fluid.

Oral Fluid Concentrations	Methylone	MDMA
Cmax	15,514.00 ± 9748.86	2936.37 ± 2761.57
Tmax	2.0 (2.0–2.0)	2.0 (2.0–4.0)
AUC_0–4 h_	40,623.79 ± 20,001.70	6586.44 ± 5229.92

Abbreviations: Area under the curve (AUC). Results of Cmax (ng/mL) and AUC_0–4h_ (ng/mL·h) are presented as mean ± standard deviation, Tmax (h) is shown as median (min–max).

## Data Availability

The data presented in this study are available on request from the corresponding author.

## Supplementary Materials

The following are available online at https://www.mdpi.com/article/10.3390/biology10080788/s1, Table S1: Summary of sociodemographic and history of substances use data of participants self-administering methylone (*n* = 8) and MDMA (*n* = 6).
