# Supplementary material for: A Comparison of Acute Pharmacological Effects of Methylone and MDMA Administration in Humans and Oral Fluid Concentrations as Biomarkers of Exposure
Source: Biology (Basel). 2021 Aug 17;10(8):788. doi: 10.3390/biology10080788 (PMC8389614; doi:10.3390/biology10080788)
Supplement: Supplementary file 1 [file biology-10-00788-s001.zip › biology-1330485-supplementary.pdf]

biology

MDPI

Table S1. Summary of sociodemographic and history of substances use data of participants self-administering methylone (n= 8) and MDMA (n= 6).

	Mean ± SD (range)	
	Methylone (n= 8)	MDMA (n= 6)
**Female/male**	3/5	1/5
**Weight (kg)**	64,9 ± 9,2 (54 - 78)	65,3 ± 8,5 (54 - 75)
**Height (m)**	1,71 ± 0,11 (1,50 - 1,86)	1,71 ± 0,12 (1,50 - 1,86)
**BMI (kg/m²)**	22,2 ± 3,5 (16,5 - 26,0)	22,4 ± 3,2 (16,5 - 25,6)
**Alcohol (units/day)**	1,0 ± 1,2 (0-3)	0,6 ± 0,9 (0 - 2)
**Tobacco (cigarettes/day)**	5,7 ± 5,0 (1 - 10) (n= 4)	8,8 ± 1,8 (7,5 - 10) (n= 2)
**Cannabis (joints/week)**	1,2 ± 0,9 (0 - 2)	1,3 ± 0,5 (1 - 2)
**MDMA**	2,3 ± 1,9 (1-5)	1,4 ± 0,5 (1 - 2)
**Methamphetamine**	0,5 ± 0,8 (0-2)	1,0 ± 1,0 (0 - 2)
**Psychostimulant NPS/ synthetic cathinones**	0,5 ± 0,8 (0 - 2)	1,5 ± 1,0 (0 - 3)
**Cocaine**	0,5 ± 1,1 (0 - 3)	0,5 ± 0,8 (0 - 2)
**Hallucinogens**	1,3 ± 1,5 (0 - 4)	1,3 ± 1,0 (0 - 3)

Abbreviations: body mass index (BMI). Frequencies of recreative substances use are expressed as daily use for alcohol and tobacco, weekly use for cannabis and previous month use for MDMA, methamphetamine, psychostimulant NPS/synthetic cathinones, cocaine and hallucinogens.
